# Supplementary material for: Release of Exosomal PD-L1 in Bone and Soft Tissue Sarcomas and Its Relationship to Radiotherapy
Source: Cancers (Basel). 2024 Jul 8;16(13):2489. doi: 10.3390/cancers16132489 (PMC11240571; doi:10.3390/cancers16132489)
Supplement: Supplementary file 1 [file cancers-16-02489-s001.zip › cancers-2732608-supplementary.pdf]

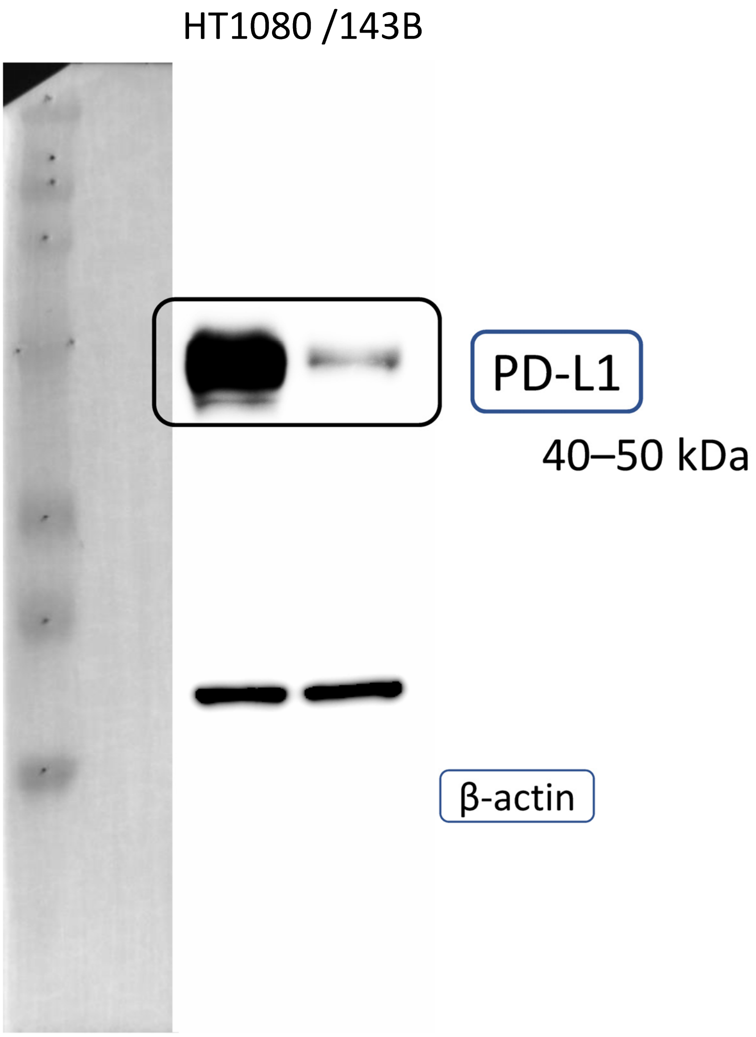

Figure S1. PD-L1 Expression in Bone and Soft Tissue Sarcoma Cell Lines.

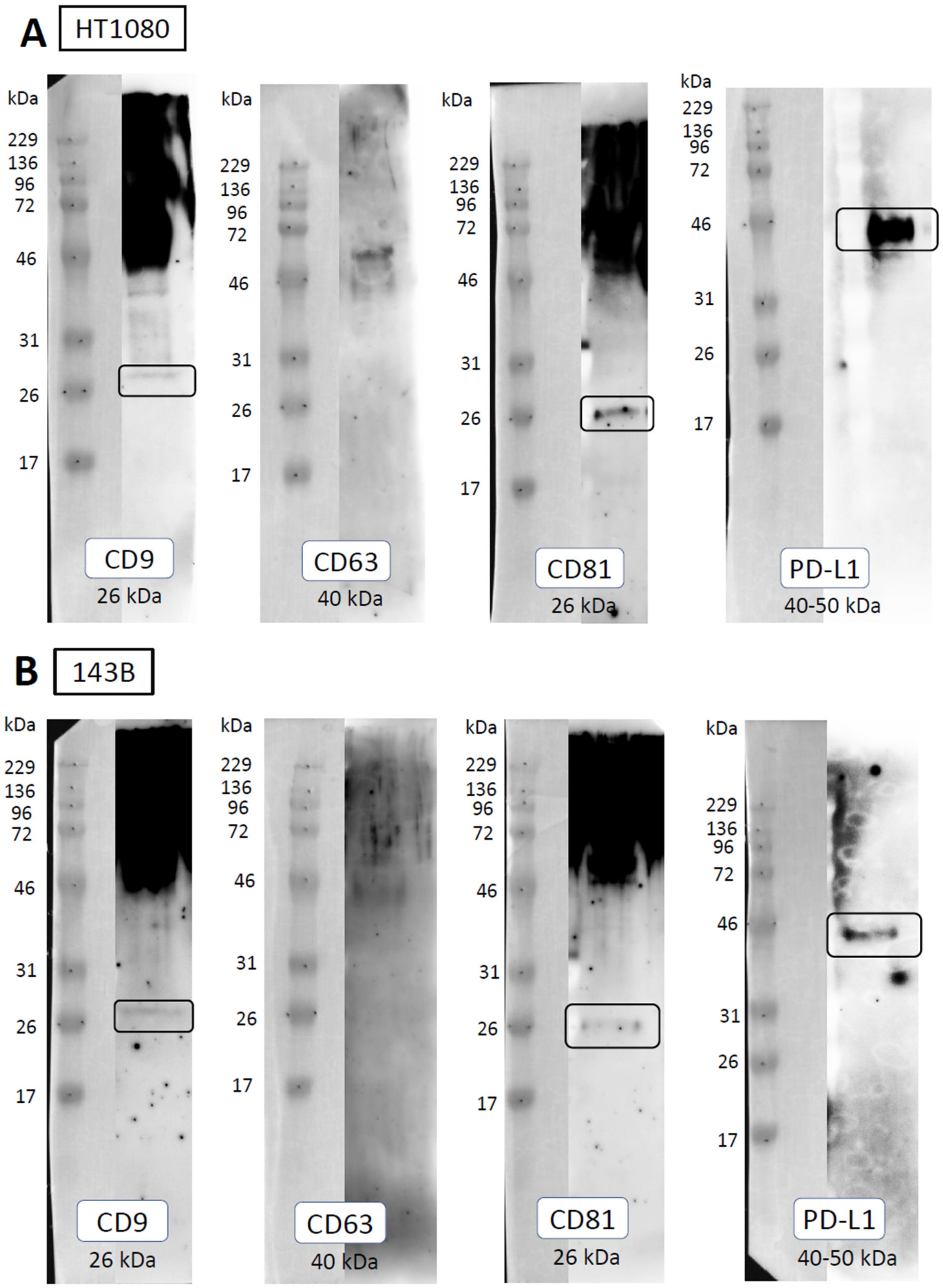

Figure S2. Exosomal PD-L1 expression in culture medium. PD-L1 is detected by Western blotting using exosomes prepared from the culture medium of HT1080 cells (A) or 143B cells (B). CD9, CD63, and CD81 are exosome markers.

A Control/HT1080/143B

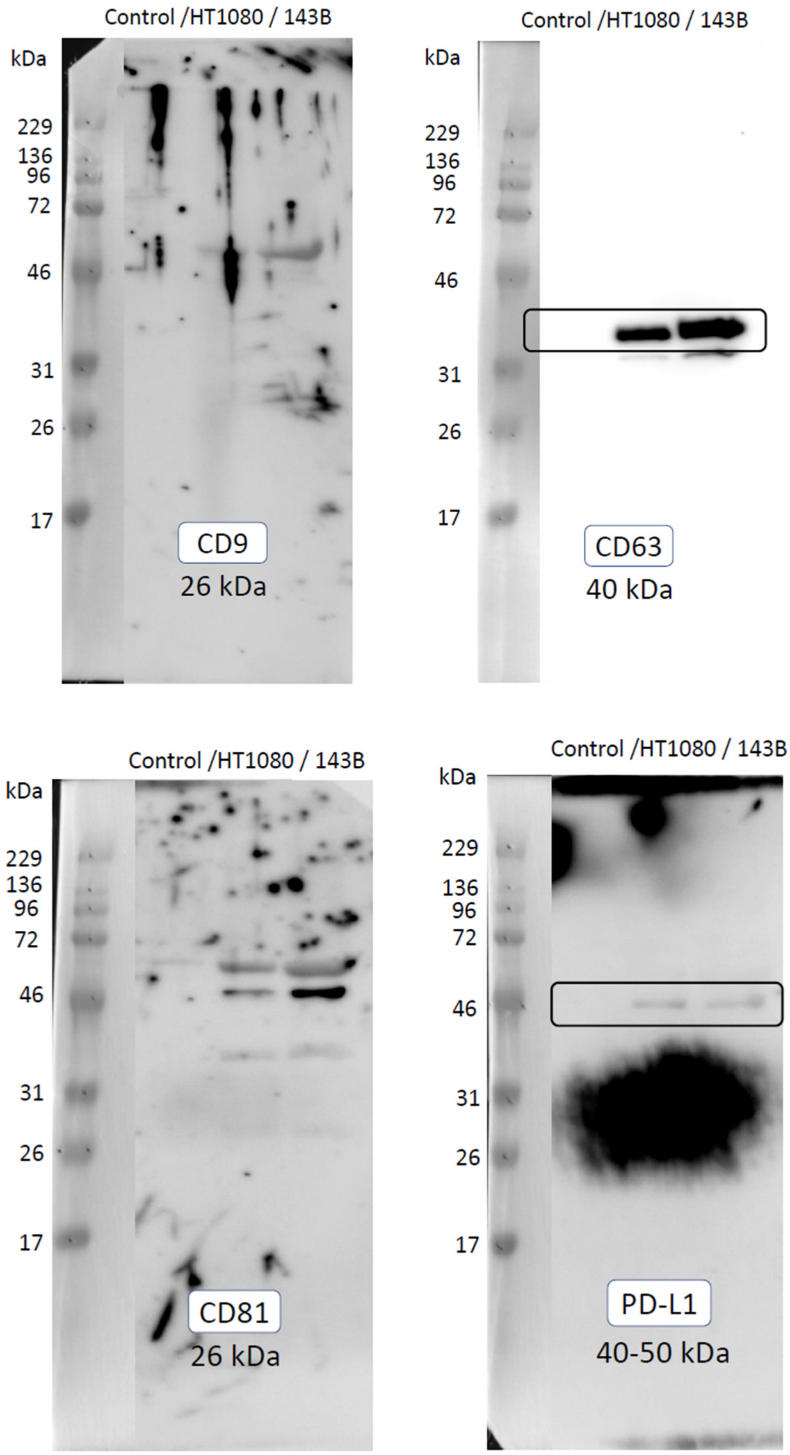

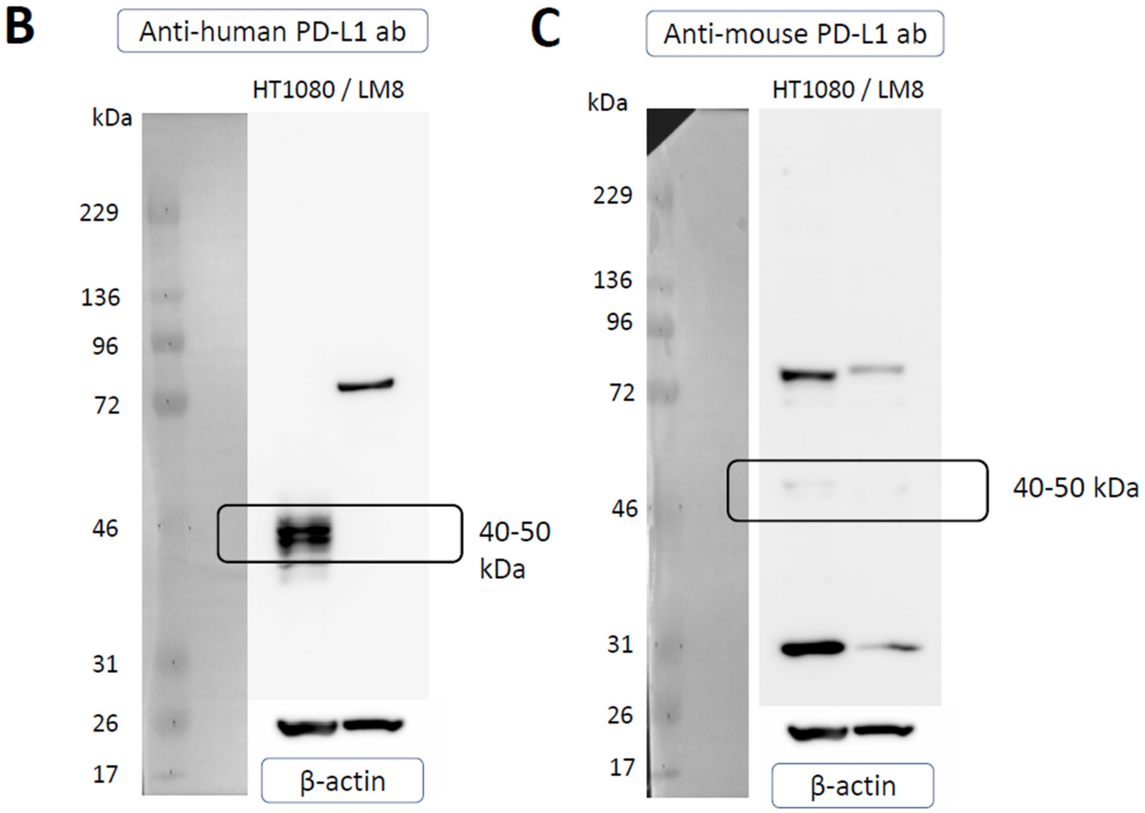

Figure S3. Exosomal PD-L1 expression in serum. Nude mice were transplanted with HT1080 cells and 143B cells. Exosomes in blood were isolated after tumor formation, and PD-L1 was detected by Western blotting. Exosome markers in serum from no tumor cell transplantation mice and human PD-L1 were not detectable (A). In mice implanted with HT1080 cells or 143B cells, CD63-positive exosome and human PD-L1 expression were detected (A). The anti-human PD-L1 antibody did not react with PD-L1 from mouse osteosarcoma cell line LM8 (B). The anti-mouse PD-L1 antibody showed around 30-, 46-, and 80 kDa band in HT1080 (human) and LM8 (mouse) cells (C).

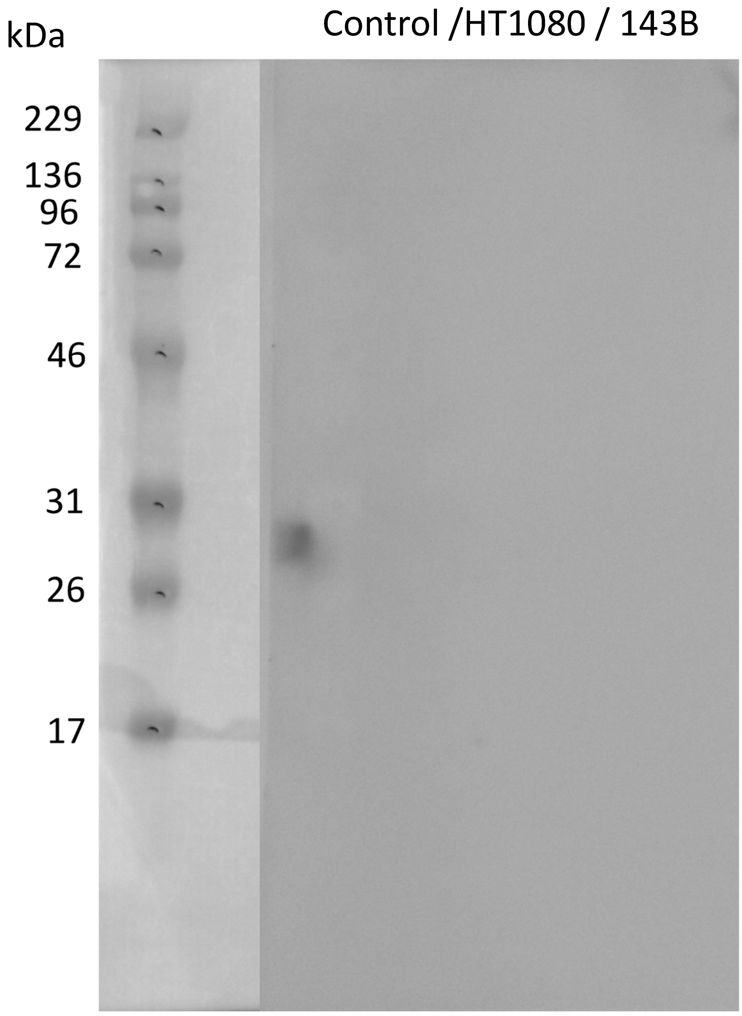

Figure S4. Reaction with secondary antibody.

A

Control	450nm
2000	1.876
1000	0.978
500	0.454
250	0.213
125	0.025
62.5	0.016
31.25	0.012
0	0.000

(pg/mL)

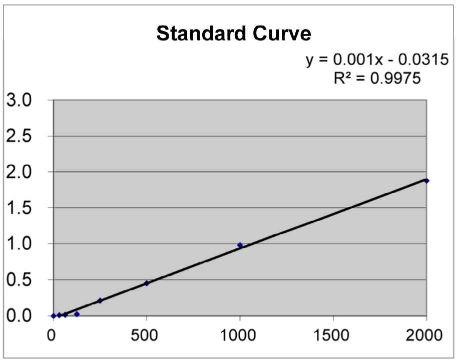

B-1

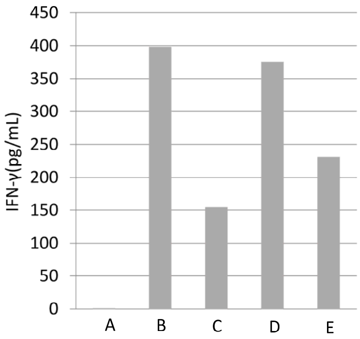

B-2

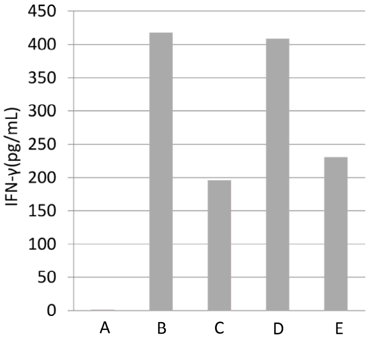

B-3

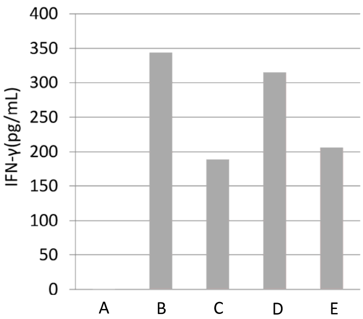

B-4

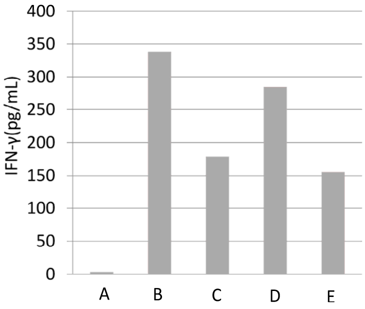

B-5

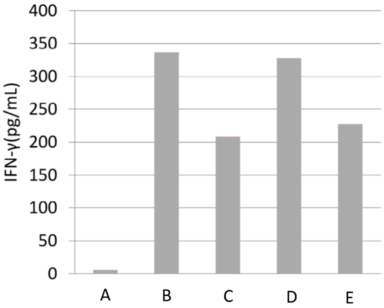

C

	IFNγ(pg/mL)				
	1	2	3	4	5
A: Control	1.06	1.484	0.742	3.5	6.363
B: PMA/Ionomycin	398.315	417.827	344.018	338.08	336.701
C: HT1080 exosome 5μL	154.299	196.082	188.659	178.902	208.383
D: HT1080 exosome 5μL/Anti PD-L1 antibody	375.62	408.495	314.855	284.95	327.899
E: HT1080 exosome 5μL/Anti control antibody	231.078	230.795	205.944	155.57	227.26

Figure S5. The standard curve and the raw data of the ELISA results. The standard curve (A) and data for each n=5 (B1–B5,C) of ELISA results were included as supplementary data.

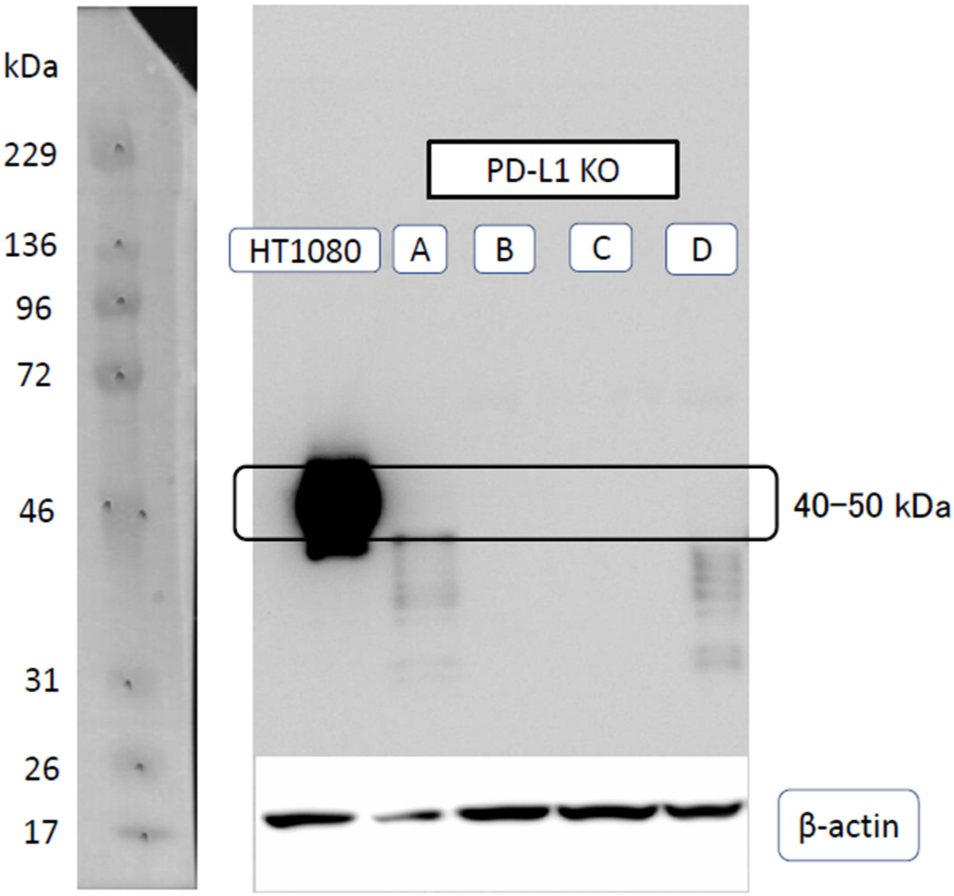

Figure S6. PD-L1 knockout HT1080 tumor cells A–D according to Western blotting. PD-L1 knockout cells were evaluated by Western blotting, which confirms the expression of cPD-L1 in HT1080 cells, whereas none of the four cells shows cPD-L1 expression (A–D).

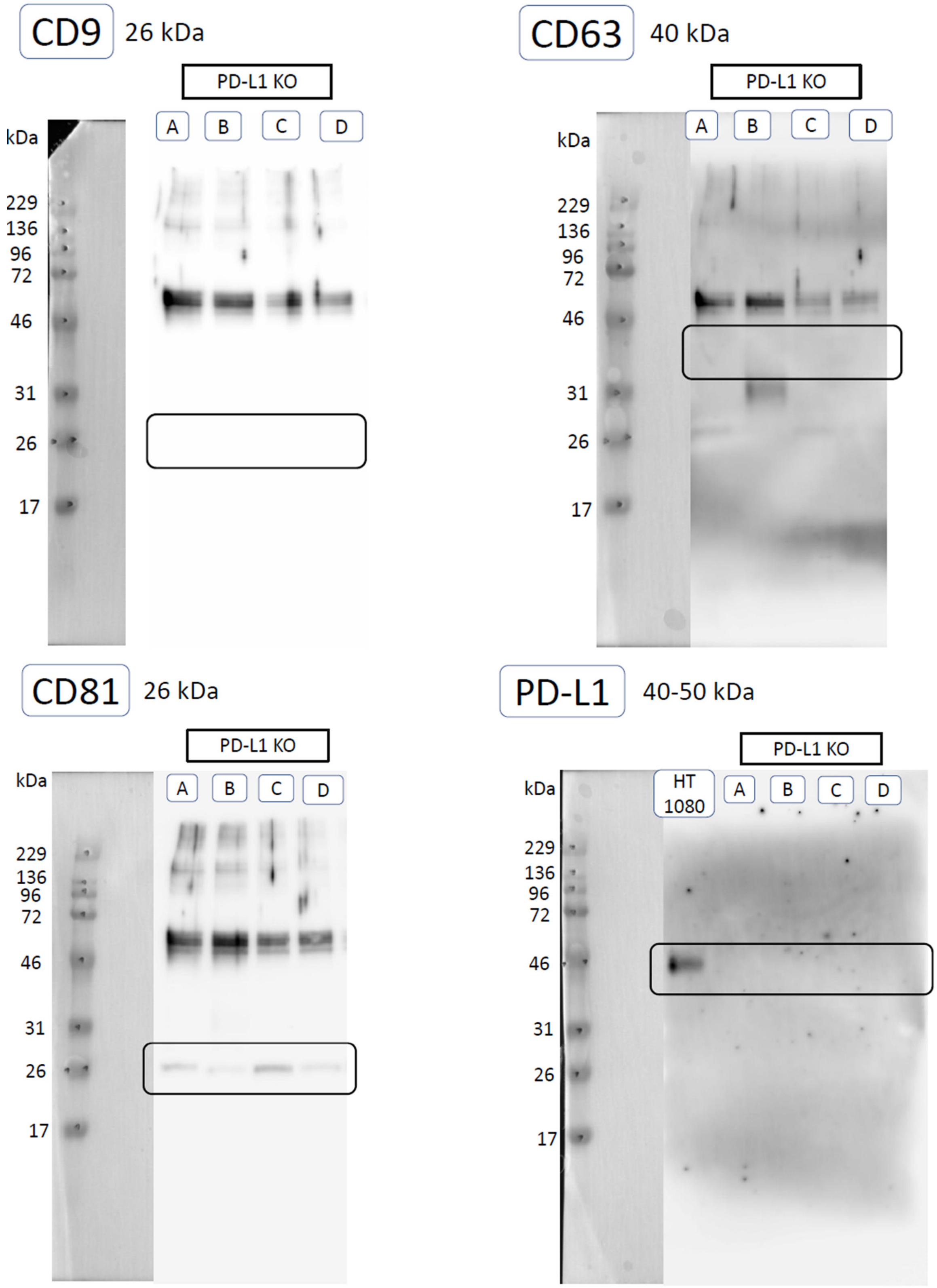

Figure S7. Exosomal PD-L1 knockout HT1080 cells on Western blotting. Exosomal PD-L1 knockout cells were evaluated by Western blotting. Exosomes were extracted from the culture medium of wild HT1080 and the four KO cells A – D, and Western blotting was performed. Exosome marker, CD81 is present in all four cells, whereas none of the four cells shows exosomal PD-L1 expression.

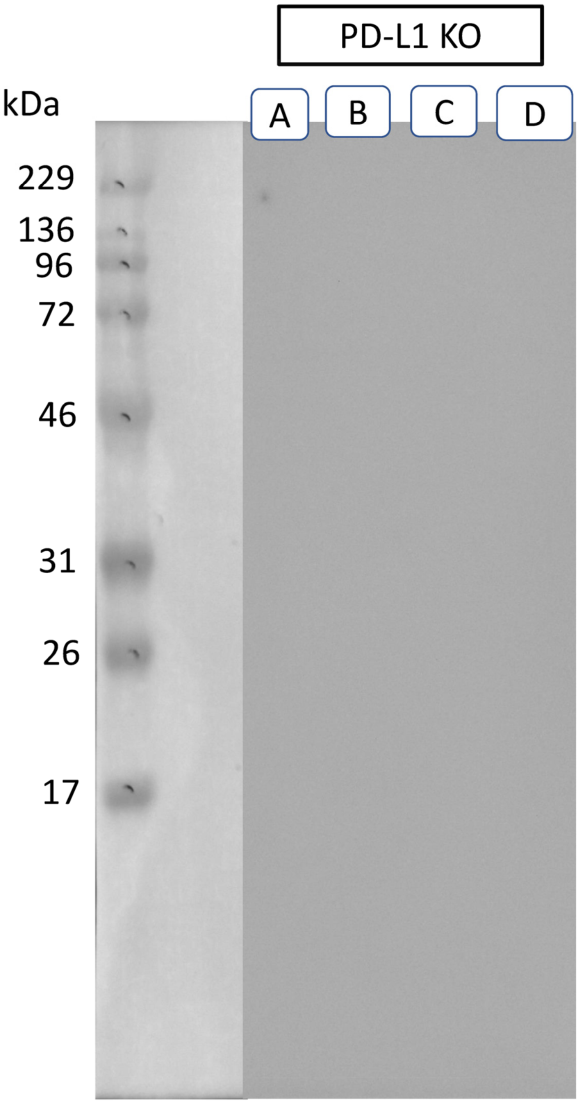

Figure S8. Reaction with secondary antibody.

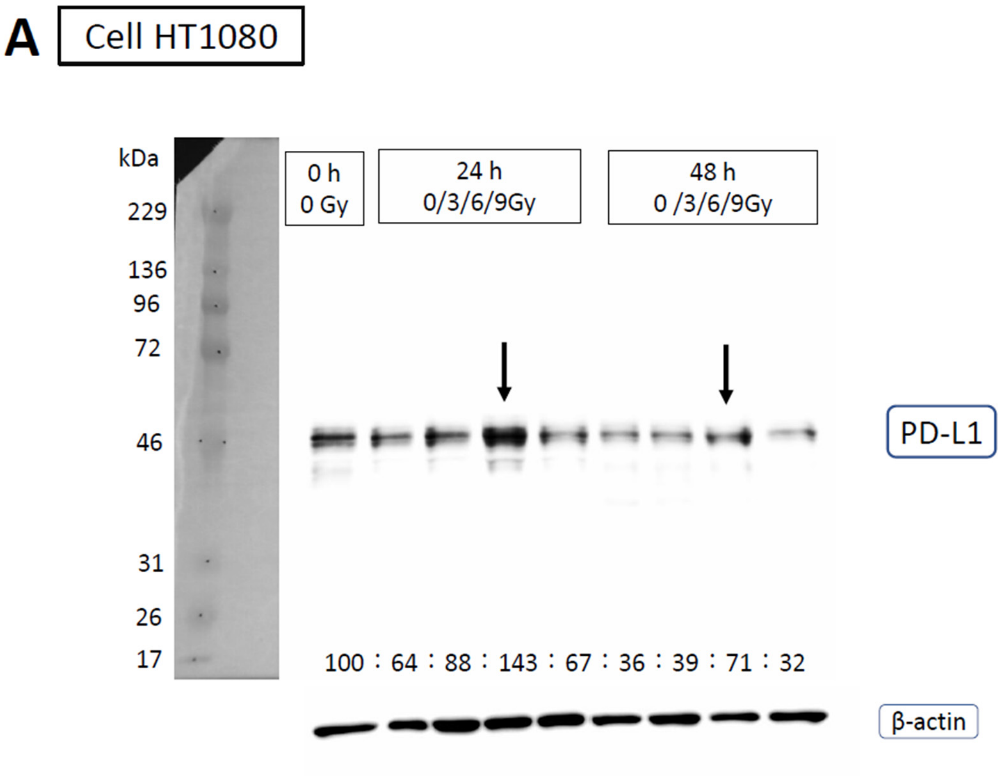
A
Cell HT1080
kDa
229
136
96
72
46
31
26
17
0 h
0 Gy
24 h
0/3/6/9Gy
48 h
0 /3/6/9Gy
PD-L1
100 : 64 : 88 : 143 : 67 : 36 : 39 : 71 : 32
β-actin

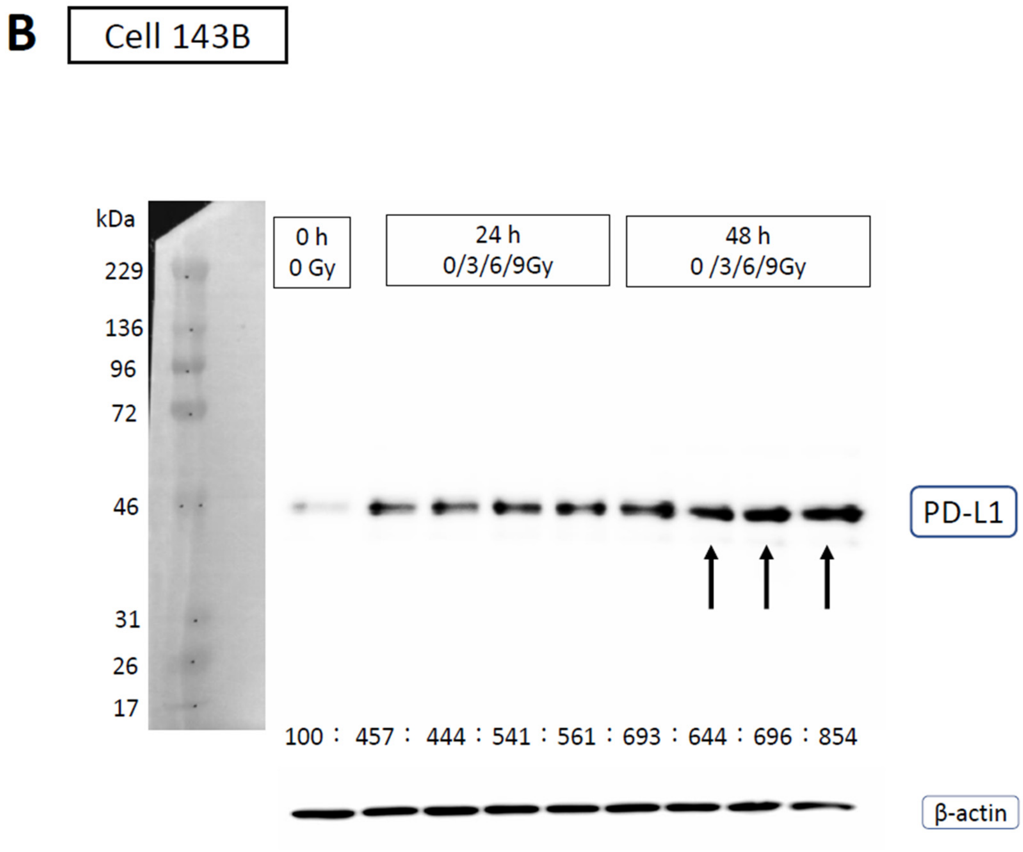
B
Cell 143B
kDa
229
136
96
72
46
31
26
17
0 h
0 Gy
24 h
0/3/6/9Gy
48 h
0 /3/6/9Gy
PD-L1
100 : 457 : 444 : 541 : 561 : 693 : 644 : 696 : 854
β-actin

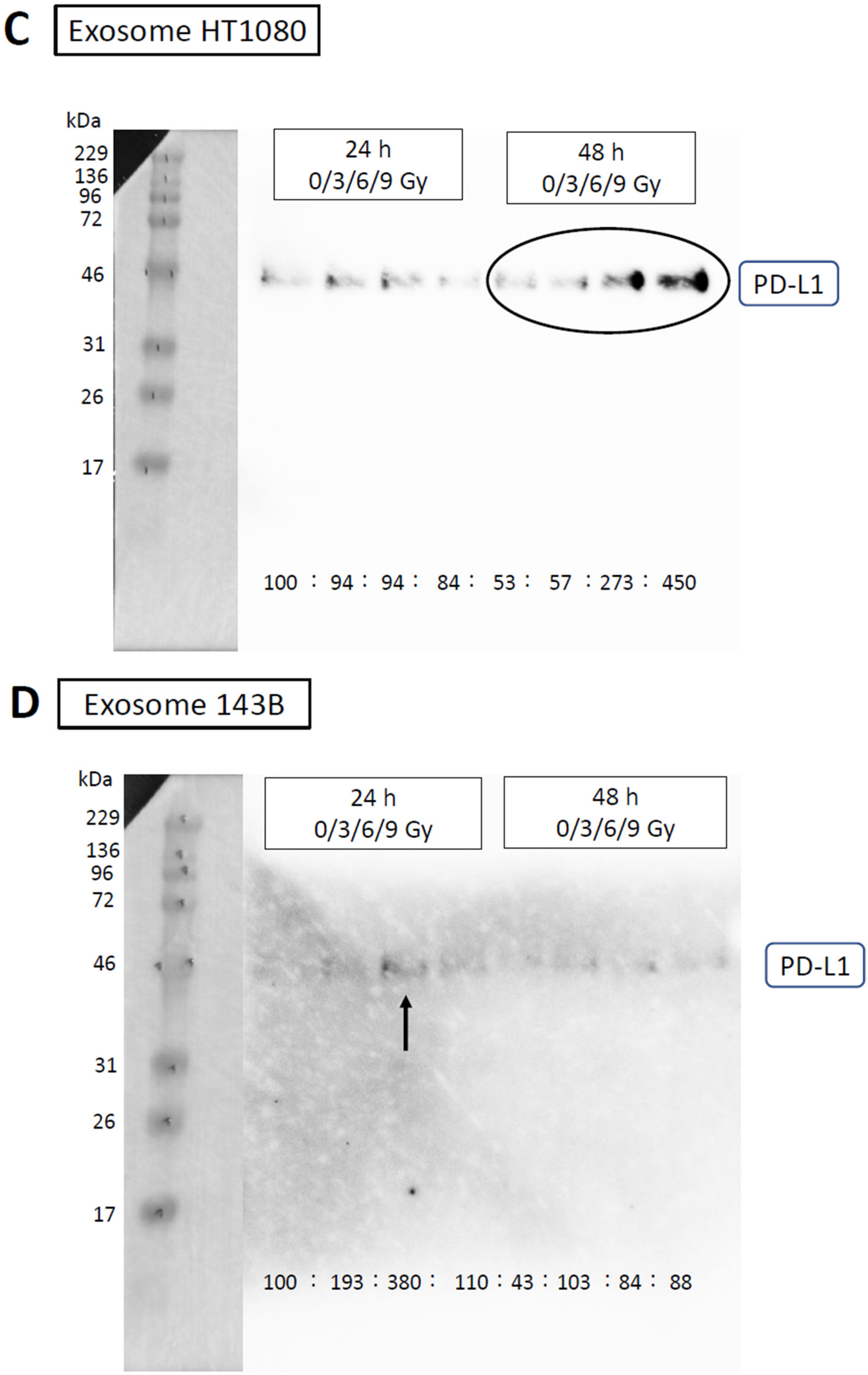

Figure S9. Effects of irradiation on PD-L1 expression in vitro. HT1080 (A) and 143B (B) cells were irradiated, and changes in cellular PD-L1 levels were detected by Western blotting. Exosomes isolated from the media of HT1080 cells (C) and 143B (D) cells were used to examine PD-L1 expression after radiation. In HT1080 cells, 6 Gy irradiation tends to increase the expression of cPD-L1 at 24 and 48 h. An intensity-dependent tendency to increase in exosomal PD-L1 protein levels is observed at 48 h

after irradiation. In 143B cells, the expression level of cPD-L1 tends to be increased in a radiation intensity-dependent manner 48 h after irradiation. Exosomal PD-L1 levels tend to be increased 24 h after 6 Gy irradiation. The arrows were marked where the bands were getting stronger or where there were changing.

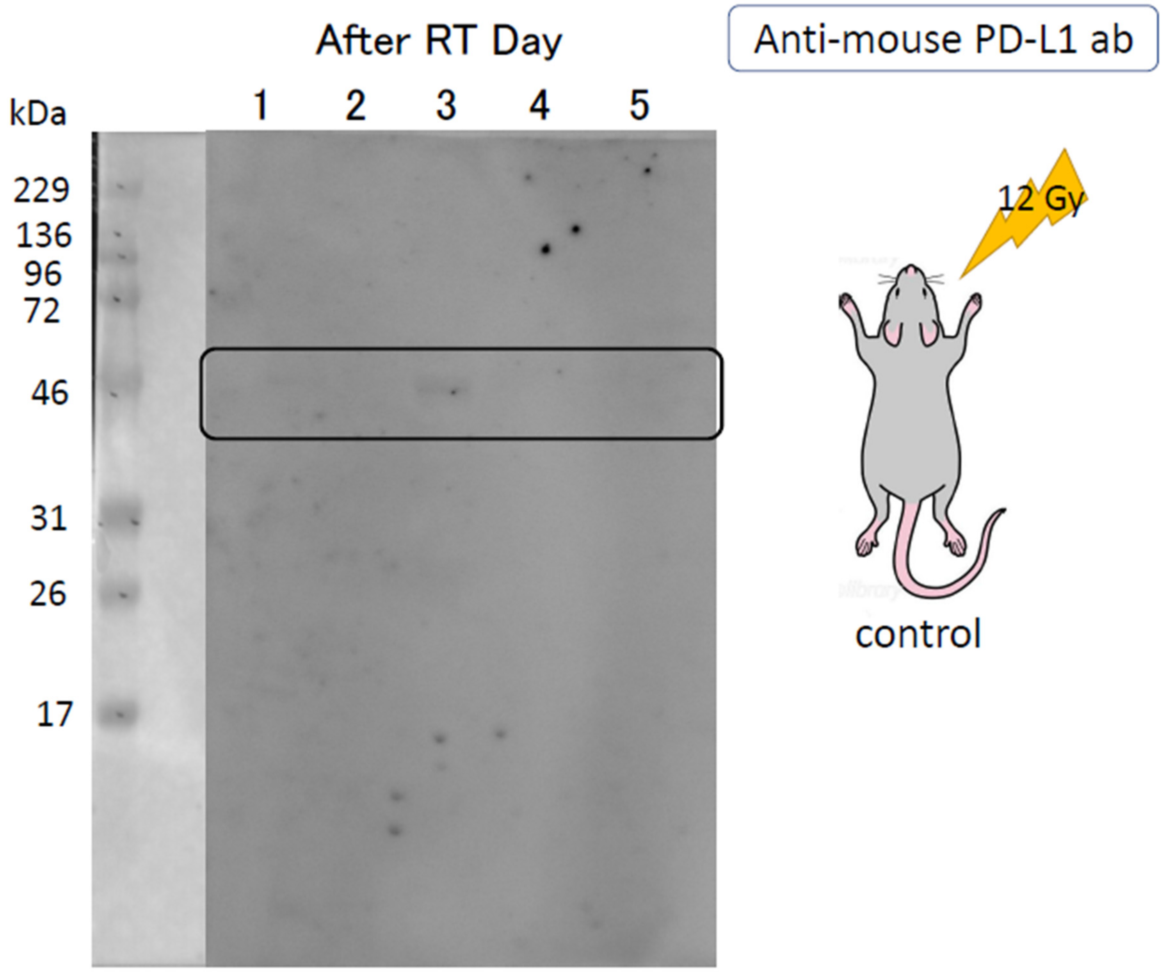

Figure S10. Effect of irradiation on the expression level of PD-L1 in individual mice. Exosomes were isolated from blood of control mice, and Western blotting was performed using an anti-mouse PD-L1 antibody. The circulating exosomal PD-L1 level is maximal three days after irradiation.

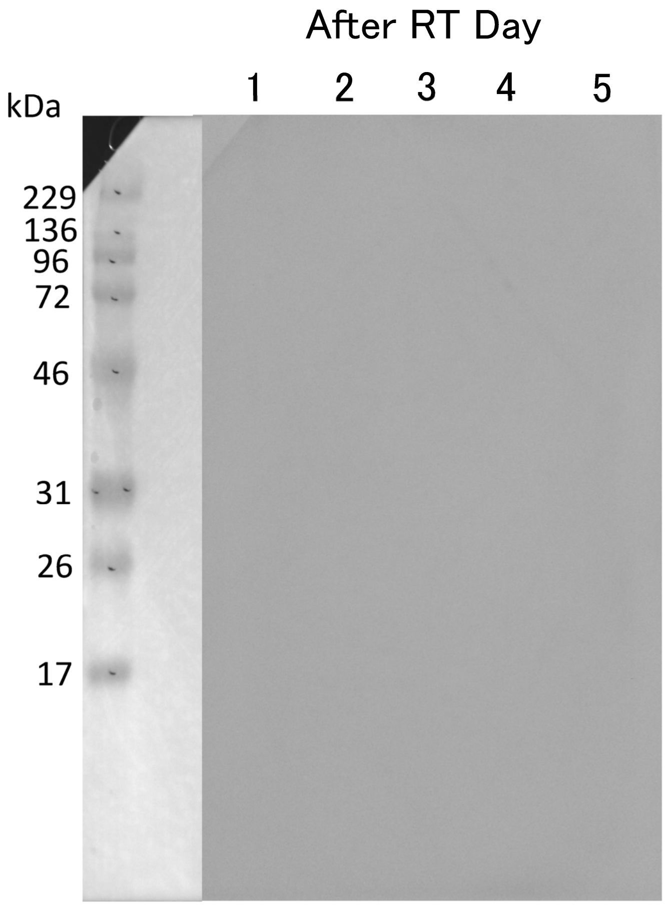

Figure S11. Reaction with secondary antibody.

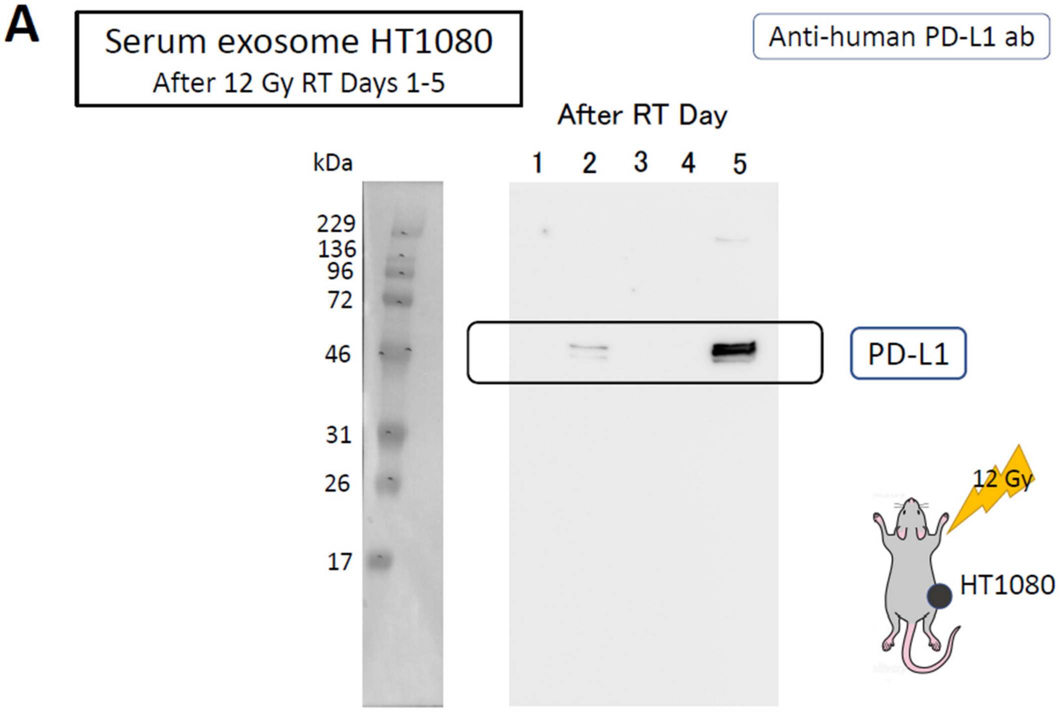

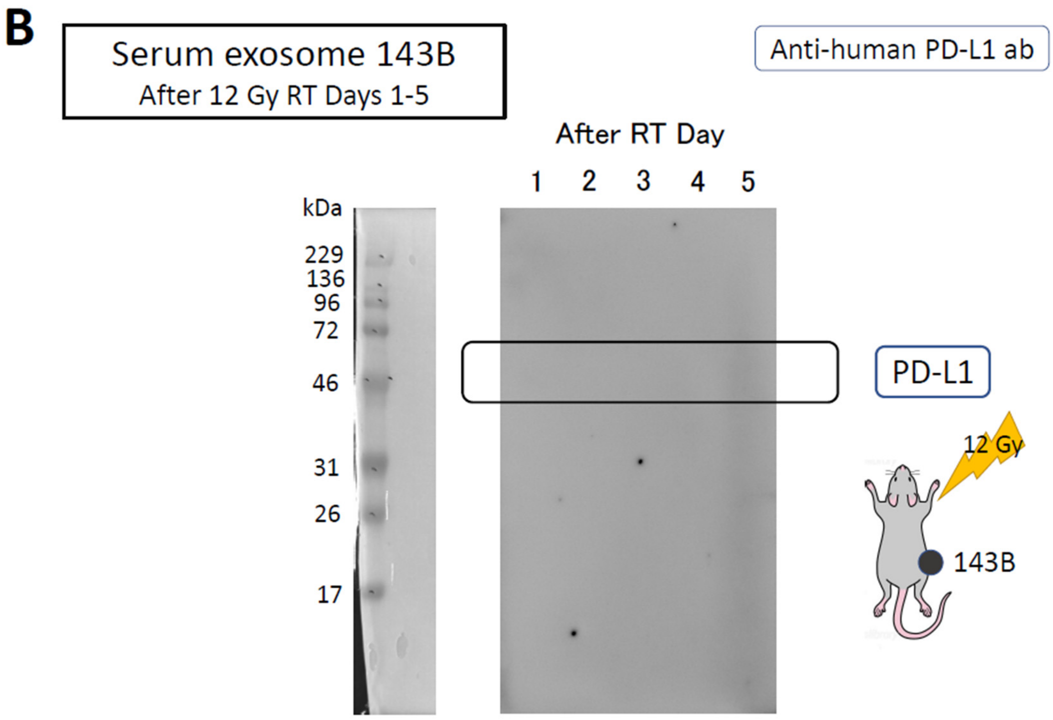

Figure S12. Effect of irradiation on the expression level of PD-L1 in individual mice. Exosomes were isolated from mice transplanted with HT1080 cells (A) and 143B cells (B), and Western blotting was performed using an anti-human PD-L1 antibody. In HT1080, human-derived exosomal PD-L1 expression strongly tends to be enhanced five days after irradiation. In contrast, no human-derived exosomal PD-L1 expression was observed in 143B cells after irradiation.

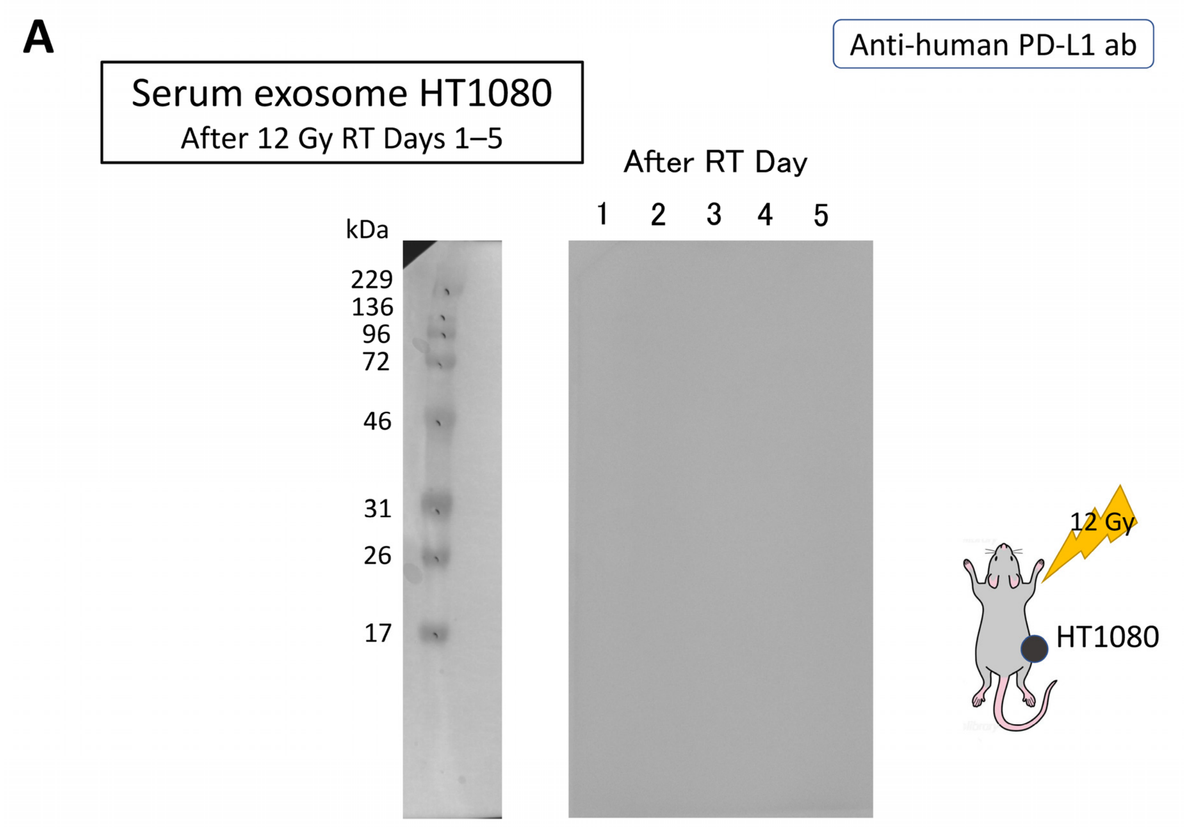

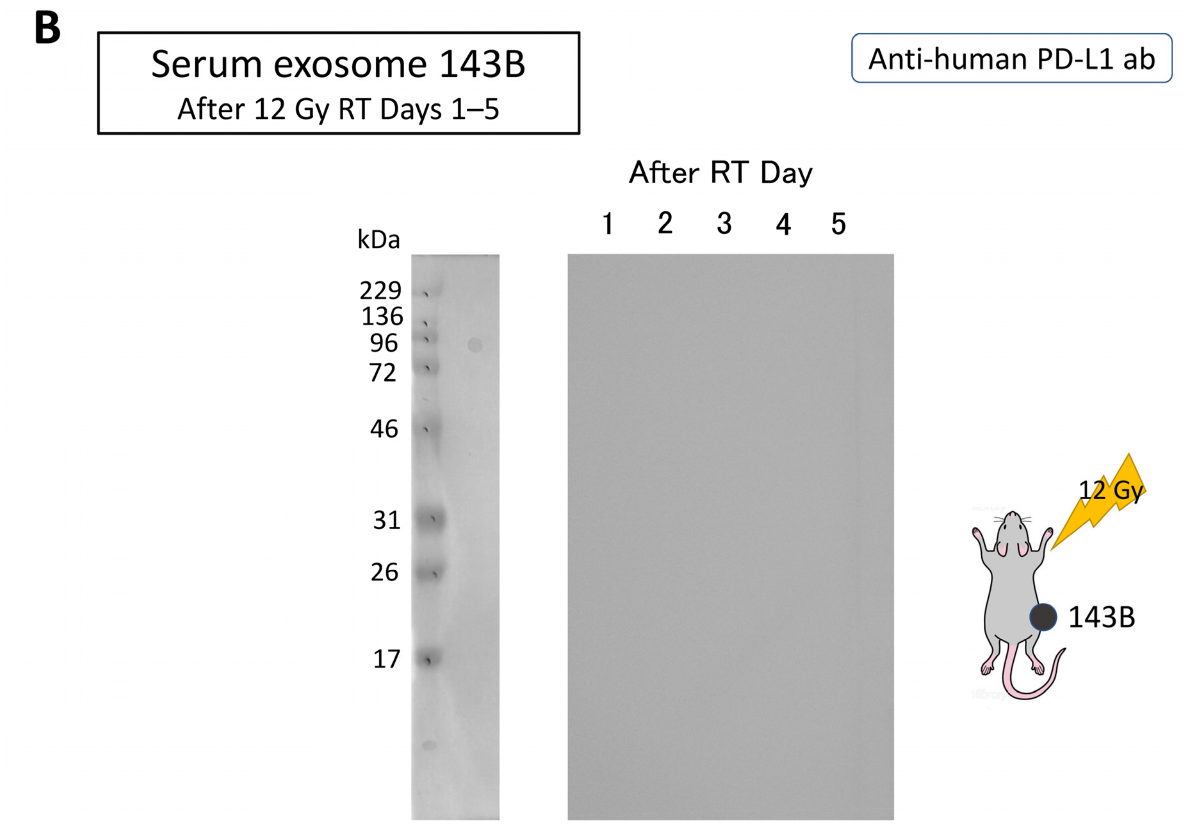

Figure S13. Reaction with secondary antibody. The samples of irradiation on mice with HT1080 (A) and 143B (B) tumors did not react with secondary antibody in western blot.
